# Anti-cancer effects of baicalein in non-small cell lung cancer in-vitro and in-vivo

**DOI:** 10.1186/s12885-016-2740-0

**Published:** 2016-09-01

**Authors:** Mary-Clare Cathcart, Zivile Useckaite, Clive Drakeford, Vikki Semik, Joanne Lysaght, Kathy Gately, Kenneth J. O’Byrne, Graham P. Pidgeon

**Affiliations:** 1 Department of Surgery, Trinity Translational Medicine Insitiute, Trinity Health Sciences Centre, Trinity College Dublin/St. James’ Hospital, Dublin 8, Ireland; 2 Department of Clinical Medicine, Institute of Molecular Medicine, Trinity Health Centre, Trinity College Dublin/St. James’ Hospital, Dublin 8, Ireland; 3 Cancer and Aging Research Program, Queensland University of Technology, Brisbane, Queensland Australia

**Keywords:** Baicalein, NSCLC, Survival, Apoptosis, Angiogenesis, in-vivo

## Abstract

**Background:**

Baicalein is a widely used Chinese herbal medicine derived from *Scutellaria baicalenesis*, which has been traditionally used as anti-inflammatory and anti-cancer therapy. In this study we examined the anti-tumour pathways activated following baicalein treatment in non-small cell lung cancer (NSCLC), both in-vitro and in-vivo.

**Methods:**

The effect of baicalein treatment on H-460 cells in-vitro was assessed using both BrdU assay (cell proliferation) and High Content Screening (multi-parameter apoptosis assay). A xenograft nude mouse model was subsequently established using these cells and the effect of baicalein on tumour growth and survival assessed in-vivo. Tumours were harvested from these mice and histological tissue analysis carried out. VEGF, 12-lipoxygenase and microvessel density (CD-31) were assessed by immunohistochemistry (IHC), while H and E staining was carried out to assess mitotic index. Gene expression profiling was carried out on corresponding RNA samples using Human Cancer Pathway Finder Arrays and qRT-PCR, with further gene expression analysis carried out using qRT-PCR.

**Results:**

Baicalein significantly decreased lung cancer proliferation in H-460 cells in a dose dependent manner. At the functional level, a dose-dependent induction in apoptosis associated with decreased cellular *f*-actin content, an increase in nuclear condensation and an increase in mitochondrial mass potential was observed. Orthotopic treatment of experimental H-460 tumours in athymic nude mice with baicalein significantly (*p* < 0.05) reduced tumour growth and prolonged survival. Histological analysis of resulting tumour xenografts demonstrated reduced expression of both 12-lipoxygenase and VEGF proteins in baicalein-treated tumours, relative to untreated. A significant (*p* < 0.01) reduction in both mitotic index and micro-vessel density was observed following baicalein treatment. Gene expression profiling revealed a reduction (*p* < 0.01) in both *VEGF* and *FGFR-2* following baicalein treatment, with a corresponding increase (*p* < 0.001) in *RB-1*.

**Conclusion:**

This study is the first to demonstrate efficacy of baicalein both in-vitro and in-vivo in NSCLC. These effects may be mediated in part through a reduction in both cell cycle progression and angiogenesis. At the molecular level, alterations in expression of *VEGF*, *FGFR-2*, and *RB-1* have been implicated, suggesting a molecular mechanism underlying this in-vivo effect.

**Electronic supplementary material:**

The online version of this article (doi:10.1186/s12885-016-2740-0) contains supplementary material, which is available to authorized users.

## Background

Lung cancer is the primary cause of cancer related death in the developed world, accounting for 12 % of deaths worldwide [1]. The majority of patients with advanced non-small cell lung cancer (NSCLC) will have a median survival of 18 months and 9 months for locally advanced or metastatic disease respectively [2]. While treatment options have improved dramatically in recent years, current therapeutic strategies remain relatively ineffective, reflected by an overall survival rate of just 15 % [3]. Non-small cell lung cancer (NSCLC) is the most common cause of cancer-related deaths in men and women, comprising approximately 80–85 % of all lung cancers [4].

Baicalein, a bioactive flavanoid, is found in extracts of the root of the plant *Scutellaria baicalensis* and has been used extensively as a Chinese herbal medicine. A range of biological effects of baicalein have been reported. It is known for its anti-inflammatory, anti-pyretic and anti-hypersensitivity properties [5], as well as demonstrating anti-viral, and anti-tumour effects. Baicalein has been previously reported to induce apoptosis in human gastric, colon, hepatoma, pancreatic and prostate cancer cells [6–10]. It has also been shown to target tumour angiogenesis and metastasis [10]. However, the mechanisms underlying these effects are poorly understood. The mechanisms underlying the effects of baicalein were previously examined in prostate and human epidermoid cancer cells, with alterations to various members of the Bcl-family of proteins, activation of the caspase cascade and PARP cleavage reported [6, 10, 11].

While the effects of baicalein on a range of human cancer cells has been investigated in-vitro, few studies have been carried out to examine its effects in-vivo. The first indication of an in-vivo growth inhibitory effect of baicalein was reported in prostate cancer [12]. A later study reported that it reduced tumour growth in hepatocellular carcinoma [8], with a further study demonstrating that it reduced the incidence of tumour formation in colitis-associated colon cancer [13]. While previous studies have demonstrated the anti-cancer efficacy of this flavanoid in NSCLC, these are based in cell lines and cannot predict the efficacy of baicalein in-vivo. Leung et al., found that baicalein inhibits tumour cells growth in NSCLC *via* induction of apoptosis. This was associated with altered regulation of cell cycle and apoptosis proteins such as bcl-2/bax, caspase-3 and p53 [14]. A more recent study carried out by Gong et al., also demonstrated dysregulation of the apoptotic machinery (bcl-2/bax ratio) as well as negatively affecting proteins implicated in angiogenesis (MMP-2, MMP-9) following baicalein treatment [5]. The negative effect on angiogenesis proteins lends support to earlier observations in human vascular endothelial cells (HUVECs) [10]. This study also demonstrated an anti-angiogenic role for baicalein in-vivo using the CAM assay.

In the current study, we examined the effect of physiologically relevant doses of baicalein on multiple pathways regulating tumour growth in NSCLC cells in-vitro and examined the use of baicalein as a therapeutic strategy in a xenograft mouse model. Using this model, we investigated the effects of baicalein treatment on tumour growth and survival in-vivo and also assessed potential mechanisms underlying these effects.

## Methods

### Cell culture and drugs

The human non-small cell lung cancer cells H-460 (large cell carcinoma), A549 (adenocarcinoma) and SKMES1 (squamous carcinoma) were obtained from the American Type Culture Collection (Rockville, MD) and maintained in a humidified atmosphere of 5 % CO_2_ in air at 37 °C. They were routinely cultured in RPMI 1640 medium, which was supplemented with 10 % (v/v) foetal bovine serum (Life Technologies Inc.), 2 μM L-glutamine, and 100 μg/ml penicillin-streptomycin. Sub-culturing was carried out when the cells reached 80 % confluency. Baicalein was obtained from Cayman Chemical (Ann Arbor, MI, USA) and made up either in DMSO (in-vitro cell culture studies) or in a solution containing 80 % PBS and 20 % DMSO (in-vivo xenograft studies). Proportionate volumes of DMSO were used for vehicle control groups in all experiments.

### Animals

Surgical procedures and care of animals was approved by the Ethics Committee of Trinity College Dublin, Ireland, and were carried out according to institutional guidelines. All experiments were carried out under a license granted by the Department of Health and Children in Ireland. Male 4–6 week old BALBc nude mice (Harlan Laboratories, UK) were housed at a constant temperature and supplied with laboratory chow and water *ad libatum* on a 12-h dark/light cycle. Mice (5/cage) were kept in isolated (with their own air supply), sterile cages in a clean facility, with bedding changed twice weekly. Animal husbandry was carried out under sterile conditions in a microbiological safety cabinet. Body weights were recorded prior to and during experimentation to ensure the ongoing health of the animals.

### Cell proliferation assay

H-460, A549 or SKMES1 cells were seeded at a concentration of 5 × 10^3^/well into 96-well plates and allowed to adhere at 37 °C overnight. Following overnight incubation in serum-deplated media (0.5 % FBS), cells were treated for 24 h with or without various concentrations (100 nM, 1 μM, 10 μM, 100 μM) of baicalein (Caymen Chemicals, Ann Arbor, MI). Serum depletion was carried out in order to closely replicate the tumour microenvironment in-vivo [15]. Thereafter, cell proliferation was assessed by a specific non-radioactive cell proliferation ELISA based on the measurement of BrdU incorporation during DNA synthesis according to the manufacturer’s instructions (Roche Diagnostics GmbH, Mannheim, Germany).

### High content screening: multi-parameter apoptosis assay

Cells were seeded in at a concentration of 5 × 10^3^/well into 96-well plates and allowed to adhere overnight at 37 °C. Following overnight incubation in serum-depleted media, cells were treated in duplicate for 24 h with 100 nM, 1 μM, 10 μM and 100 μM baicalein. A positive apoptosis control treatment (10 μM cisplatin) was also used. Parameters relating to the process of apoptosis was then analysed using the Multi-parameter Apoptosis 1 HitKit (Cellomics Inc, Pittsburgh, PA, USA) following the manufacturers’ instructions. Briefly, 30 min prior to completion of the compound incubation, 50 μL of MitoTracker/Hoescht solution was added to each well and incubated at 37 °C for 30 min. 100 μL of pre-warmed fixation solution (7.3 mL of 37 % formaldehyde added to 14.7 mL 1X Wash Buffer) was then added directly to each well and the plate was incubated in a fume hood at RT for 10 min. The wells were then washed in 1X Wash Buffer, and 1X Permeabilization Buffer was added for 90 s. Following a further washing step, 50 μL AlexaFlour Phalloidin Solution was added to each well and the plates incubated for 30 min. The plates were washed 3 times in 1X Wash Buffer, with the last wash left in the wells. Plates were then sealed and analysed on the InCell 1000 Analyser (GE/Amersham Biosciences, Piscataway, NJ, USA), according to manufacturers’ instructions (Cellomics Inc., Pittsburgh, PA, USA). Analysis of the 96-well plates was carried out by a trained user of the InCell analyser software.

### Xenograft mouse model: assessment of the effects of baicalein on tumour growth and survival in-vivo

H-460 cells (1 × 10^6^) were administered subcutaneously into the left dorsal flank of 6-week-old male nude mice (BALBc). When tumour size reached approximately 50 mm^3^, animals were randomised (blindly) into control and treatment groups (*n* = 7/group). Mice were administered either the flavanoid/LOX inhibitor, baicalein (1 mg/kg or 3 mg/kg in 50 μl DMSO/PBS), or an equal volume of a vehicle control (20 % DMSO in PBS), by intratumoural injection (3 groups in total; each group represents an experimental unit). Intratumoural injection was carried out twice weekly, and tumour size was measured every 48 h using a digital callipers. Tumour volume was calculated from size measurements using the formula *V* = width × length × Π/6. Body weights were recorded at the beginning of the experiment and subsequently at all intervals where tumour size was recorded. Animals were regularly monitored for evidence of any adverse experimental effects (such as dramatic weight loss or tumour ulceration), although none were observed. Experiments were terminated when tumours reached a size of 1500 mm (in any direction) and the animals were sacrificed by cervical dislocation. Tumours were then isolated and excised for further analysis. A portion of the tumour was placed in formalin, processed, and embedded in paraffin for histological analysis. The remaining portion was removed into RNAlater® (Qiagen, Sussex, UK) overnight (at 4 °C) before storing at −80 °C for RNA analysis.

### Gene expression analaysis following baicalein treatment in-vitro and in-vivo: qPCR arrays

Gene-expression profiling was carried out on tumour tissue isolated from the sub-cutaneous xenograft model of tumour growth (previously described). Briefly, total RNA was extracted from tumour tissue samples using a Qiagen RNeasy® Mini Kit, according to manufacturers’ instructions (Qiagen, Sussex, UK). A DNase treatment step was also included in this protocol to ensure the highest RNA quality. First strand cDNA was synthesized using the ReactionReady™ First Strand cDNA synthesis kit (Molecular Research Center Inc., OH, US), as previously described. Gene expression profiles following baicalein treatment in the H-460 cell line in-vivo were assessed by quantitative PCR array, using the RT^2^Profiler™ PCR Array Human Cancer PathwayFinder (SuperArray Bioscience Corporation, MD, US) (*n* = 2 pooled control samples and 2 pooled 1 mg/kg baicalein samples). Quantitative RT**-**PCR was carried out in all groups for the expression of a panel of genes of interest following baicalein treatment (selected from PCR-array results data and also based on previous observations in the literature). Genes of interest included *VEGFA*, *FGFR2*, *ITGAV*, *BCl-2*, *MMP-2*, *MMP-9*, *IGF-1* and *Ang-2*. This qRT-PCR was carried out using validated primer/probe sets (Life Technologies, Applied Biosystems, Carlsbad, CA, USA) and was run on a 9500 thermal cycler (Applied Biosystems, Life Technologies). 18S was used as an endogenous control for data normalization. Analysis was performed using SDS 2.3 and SDS RQ 1.2 relative quantification software (Applied Biosystems). One untreated (vehicle-treated) sample was set as the calibrator for analysis.

In a separate set of experiments, the A549 and SKMES1 cells were cultured in 6-well plates and serum depleted overnight. Thereafter cells were treated with 1 μM baicalein for 24 h and RNA was extracted using a Qiagen RNeasy® Mini Kit, according to manufacturers’ instructions (Qiagen, Sussex, UK). cDNA was prepared as described above and gene expression profiling carried out using Taqman quantitative PCR arrays (Cancer Profiler Arrays, Superarray). Genes listed were found to be differentially regulated (greater than 2 fold increase/decrease) in the baicalein-treated cells, relative to vehicle-treated controls.

### Histological analysis following baicalein treament in-vivo

Histological analysis was also carried out on all tissue samples isolated from mouse xenografts. 5 μM sections were cut from all paraffin blocks and stained for 12-LOX, VEGF and CD-31 (as microvessel density marker). Heamatoxylin and eosin staining was carried out to assess mitotic cell activity/mitotic index. Immunohistochemical staining was carried out manually using Vectastain Elite Kits (Vector labs, Burlingame, CA, USA) and rabbit polyclonal IgGs specific for 12-LOX (1:200; American Diagnostica, Stamford, CT, USA), VEGF (1:500; Millipore, Billerica, MA, USA), and CD-31 (1:100 DAKO, Glostrup, Denmark). Sections were incubated in the primary antibody for 1 h at room temperature. Staining was visualized and quantified using a Nikon 900i light microscope.

CD-31 microvessel density quantification was carried out by manually counting the number of vessels in each high-powered field of view under x 20 magnification (variation in xenograft sizes between groups), with the average number of vessels then calculated for each xenograft sample. Quantification was carried out by 3 independent observers. Mitotic index was estimated using a 1 mm^3^ grid, counting an average of 500 tumour cells per mm^3^. 10 fields were scored by 2 independent observers (Z.U., C.D.) in a blinded fashion. Mitotic cells were identified morphologically and the mean number of mitotic cells in 10 fields used as the mitotic index.

### Statistical analysis

Statistical comparison between treatments was carried out using ANOVA with post-test analysis by Tukey-Kramer multiple comparisons test. Data are taken as significant where *p* < 0.05. Statistical comparison of groups (as unit of measurement) was carried out using a 2-tailed Student’s *t-test* or ANOVA with Scheffe post-hoc correction**.** Results are expressed as mean ± SEM. Data were taken as significant where *p* < 0.05. Statistical analysis was carried out using GraphPad Prism 5.0 (GraphPad Software Inc., La Jolla, CA, USA).

## Results

### Effect of baicalein treatment on lung cancer cell survival

**Fig. 1:**
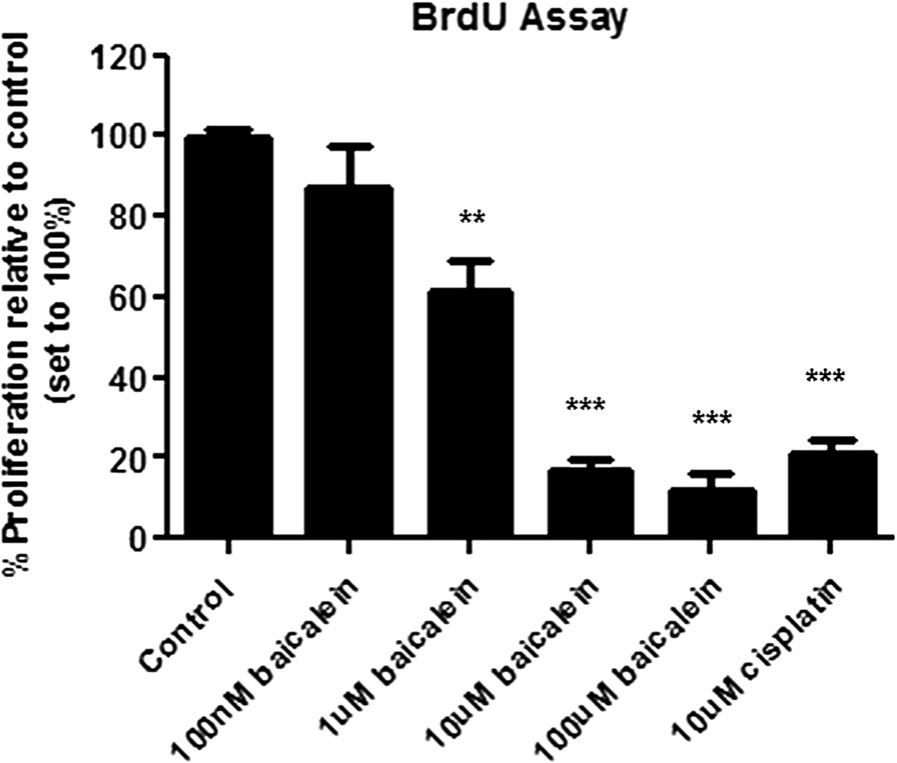
Effect of baicalein treatment on lung tumour cell proliferation/survival. Tumour cell proliferation was assessed following 24 h treatment (100 nM, 1 μM, 10 μM and 100 μM baicalein) by BrdU assay. Baicalein treatment resulted in a significant reduction in tumour cell survival in H-460 cells. Data is expressed as mean ± SEM of three independent experiments, with cell proliferation expressed as a percentage of untreated controls (*p < 0.05, **p < 0.01, ***p < 0.0001)

The flavanoid, baicalein induced a significant growth inhibition in lung cancer cells in a dose-dependent manner as measured by BrdU incorporation into H-460 cells at 24 h, relative to control cells (Fig. 1). This growth inhibition was first observed at 1 μM baicalein (61 ± 8.9 % baicalein *vs.* 99 ± 2.5 % untreated; *p* < 0.01) and further exacerbated following treatment with both 10 μM (17 ± 2.9 % baicalein *vs.* 99 ± 2.5 % untreated; *p* < 0.0001) and 100 μM baicalein (12 ± 4.5 % baicalein *vs.* 99 ± 2.5 % untreated; *p* < 0.0001). Treatment with 10 μM of the positive anti-neoplastic agent, cisplatin resulted in a similar anti-proliferative effect (21 ± 3.74 % cisplatin *vs.* 99 ± 2.5 % untreated; *p* < 0.0001).

To demonstrate that the effect of baicalein was not unique to H460 cells, A549 cells representing adenocarcinoma and SKMES1 cells (squamous carcinoma) were also treated with baicaelin and baicalein significantly reduced proliferation of each of these NSCLC subtypes (Additional file 1: Figure S1). These data show that baicalein has broader applicability as an anti-cancer agents across various NSCLC subtypes.

### Induction of cell death following baicalein treatment

**Fig. 2:**
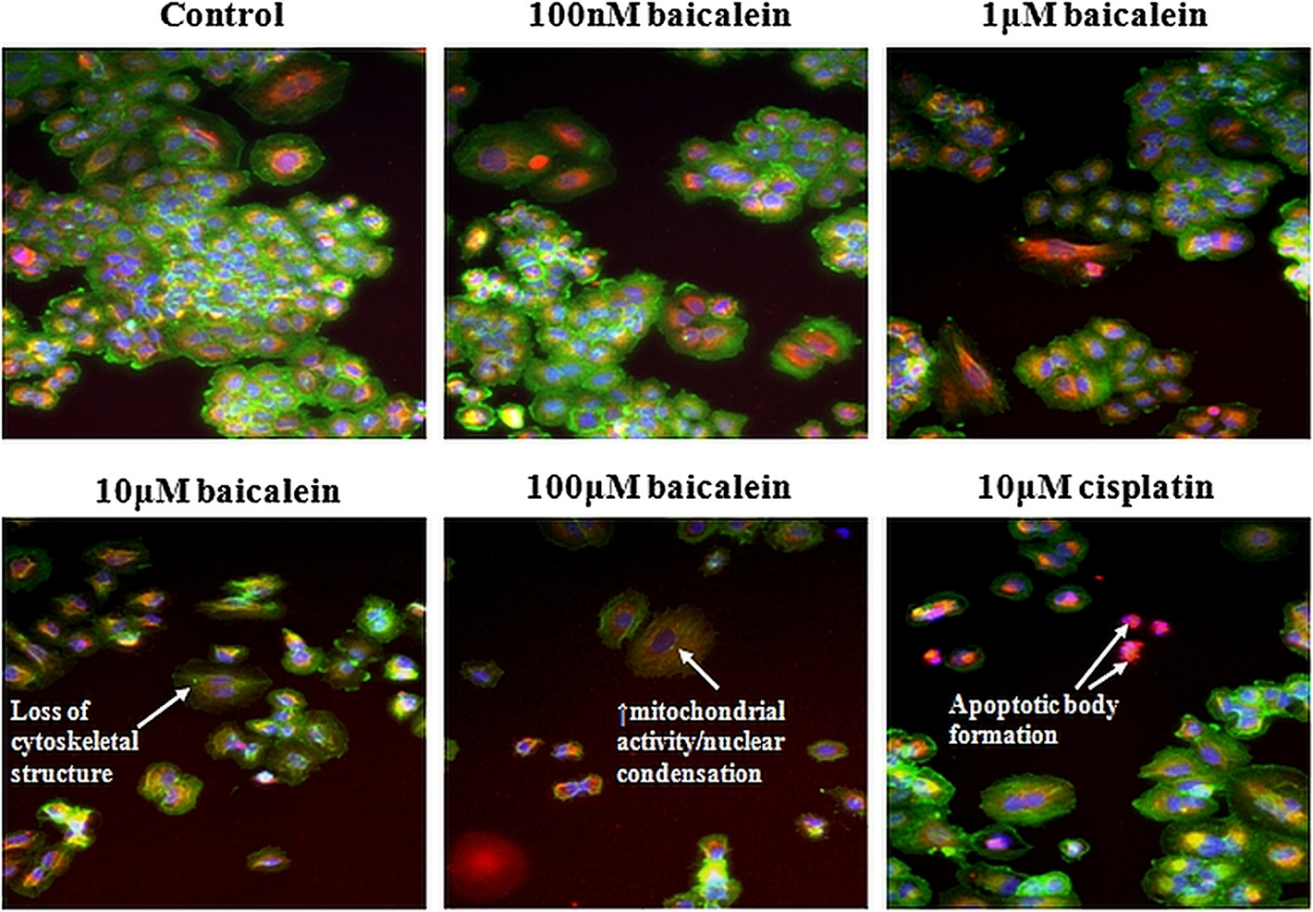
Multi-parameter apoptosis analysis of baicalein-treated H-460 cells. Morphologic features of apoptosis were identified in-vitro by High Content Screening analysis. Apoptosis was induced in a dose-dependent manner after treatment with 100 nM, 1 μM, 10 μM and 100 μM concentrations of baicalein when compared to control cells. 10 μM cisplatin was used as a positive apoptosis control. 3 spectrally distinct fluorophore labels were used to assess cell health by examining nuclei, *f*-actin (cytoskeletal protein) and mitochondrial potential. Loss of *f*-actin (*green*) shows the loss of cell integrity during apoptosis as membrane blebbing occurs and mitochondrial activity increases during apoptosis (*orange*) coupled with an increase in nuclear condensation

A dose-dependent induction of apoptosis following baicalein treatment was observed in H-460 cells. High Content Screening analysis was carried out following 24 h baicalein treatment. Multi-parameter analysis of morphological features of apoptosis was assessed using the GE In Cell Analyser. Three spectrally distinct fluorophore labels were used to examine fundamental parameters of apoptosis; loss of *f*-actin content (cytoskeletal integrity), increased nuclear condensation and increased mitochondrial mass/potential (Fig. 2). A reduction in Alexa Flour®488Phalloidin staining corresponded with loss of *f*-actin and thus a loss of cell integrity, a hallmark of apoptosis. This was evident at 10 μM baicalein treatment, and more pronounced at 100 μM when compared to untreated control cells (Fig. 2a). Nuclear condensation and fragmentation, viewed with aid of Hoescht staining of the nuclei, was observed in cells treated with baicalein, compared with untreated cells, which have intact normal-sized nuclei. An increase in Mito Tracker® Red staining also occurred in treated cells (also evident at 10 μM and 100 μM concentrations) when compared to controls. This corresponded to an increase in mitochondrial activity, coupled with a loss in potential across the mitochondrial membrane, and also occurs during apoptosis.

**Fig. 3:**
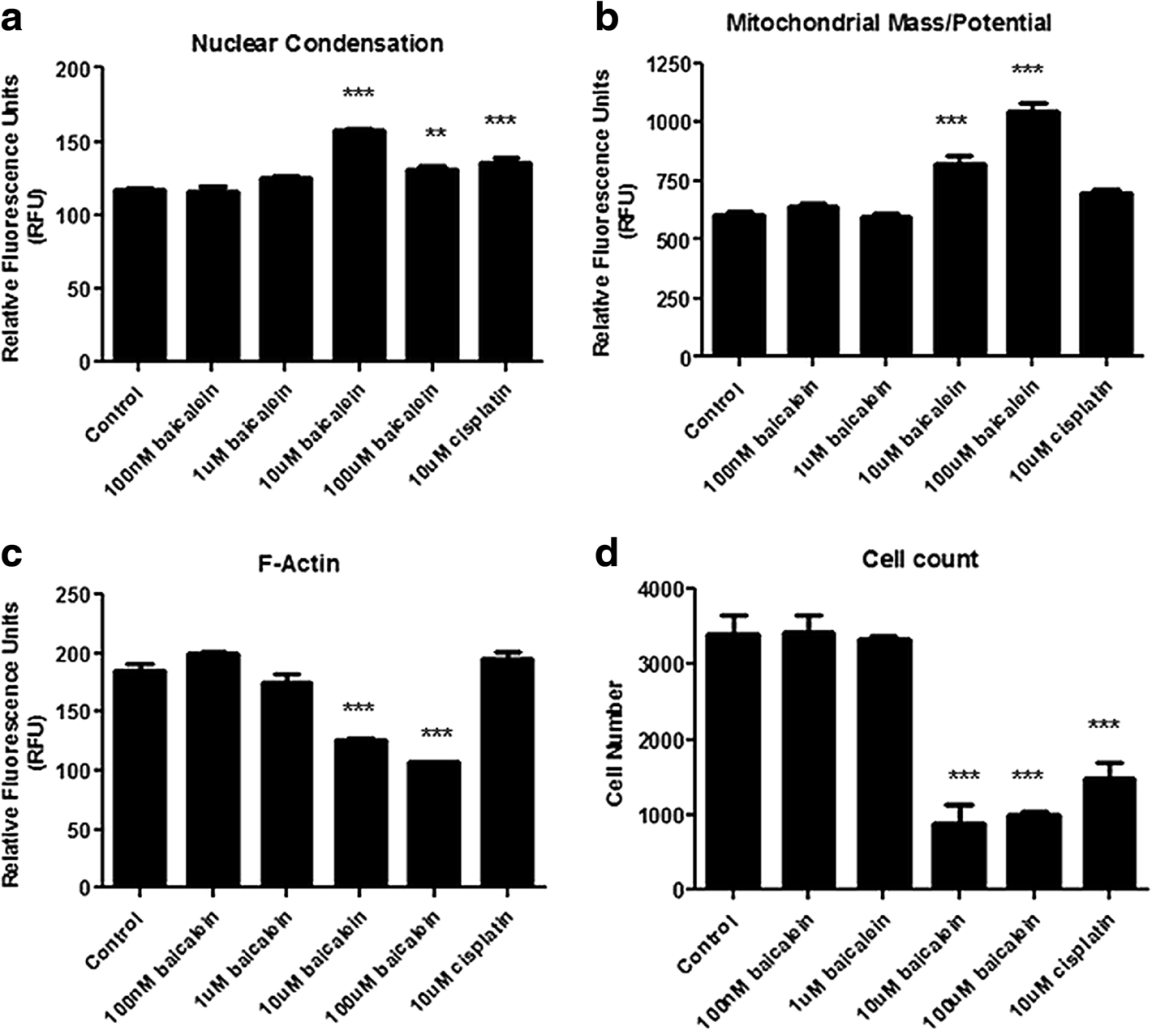
Quantification of morphologic features of apoptosis following baicalein treatment. The In Cell Analyser was used to quantify apoptosis markers following treatment with increasing concentrations of baicalein (100 nM, 1 μM, 10 μM and 100 μM) and High Content Screening. Levels of *f*-actin were significantly reduced by baicalein (**a**), while nuclear condensation (**b**) and mitochondrial mass/potential (**c**) were both increased. Cell count was also significantly reduced following treatment (**d**), confirming earlier observations. Data is expressed as mean ± SEM of three independent experiments (**p < 0.01, ***p < 0.0001)

Quantification of multi-parameter apoptosis signalling was carried out using In Cell Analyser Software, confirming qualitative observations. Baicalein treatment resulted in a significant (*p* < 0.0001) reduction in *f*-actin content (Fig. 3a) with a significant increase in both nuclear condensation (Fig. 3b; *p* < 0.0001) and mitochondrial mass/potential (Fig. 3c; *p* < 0.0001) also observed. The reduction in *f*-actin content was apparent at 1 μM concentration (175 ± 9.6 units 1 μM baicalein *vs.* 185 ± 8.6 units untreated), but reached statistical significance following treatment with 10 μM (126 ± 1.72 units) and 100 μM (107 ± 0.4 units) of the drug. Treatment with cisplatin had no effect on cytoskeletal integrity (195 ± 6 units). The increase in nuclear condensation observed following treatment only reached significance at 10 μM concentration (157 ± 1.9 units 10 μM baicalein *vs.* 117 ± 1.5 units untreated), an effect that was maintained at 100 μM (131 ± 1.6 units; *p* < 0.01). A similar significant increase in fragmentation was also observed following cisplatin treatment (136 ± 1.6 units 10 μM cisplatin *vs.* 117 ± 1.5 units untreated). Mitochondrial activity (mass/potential) was similarly increased following baicalein treatment, an effect that reached significance at 10 μM (824 ± 41.1 units 10 μM baicalein *vs.* 603 ± 22.5 units untreated) and 100 μM (1043 ± 44.3 units) concentrations. As with *f*-actin, no change in mitochondrial activity was seen following cisplatin treatment (697 ± 17 units 10 μM cisplatin *vs.* 603 ± 22.5 units untreated).

Cell number was also recorded following baicalein treatment using the In Cell Analyser. At concentrations of 10 μM and 100 μM (Fig. 3d), a drastic reduction in cell number can be seen compared to control cells and cells treated with the other two concentrations (891 ± 286.6 cells 10 μM baicalein *vs.* 3414 ± 300 cells untreated; 989 ± 89.4 cells 100 μM baicalein *vs.* 3414 ± 300 cells untreated; *p* < 0.0001). This was comparable with the cell count observed following cisplatin treatment (1489 ± 256.7 cells 10 μM cisplatin *vs.* 3414 ± 300 cells untreated; *p* < 0.001), with baicalein treatment demonstrating an even greater effect on cell number. These findings support the findings of the proliferation assays, reported in Fig. 1.

### The effect of baicalein on tumour growth and survival in-vivo

**Fig. 4:**
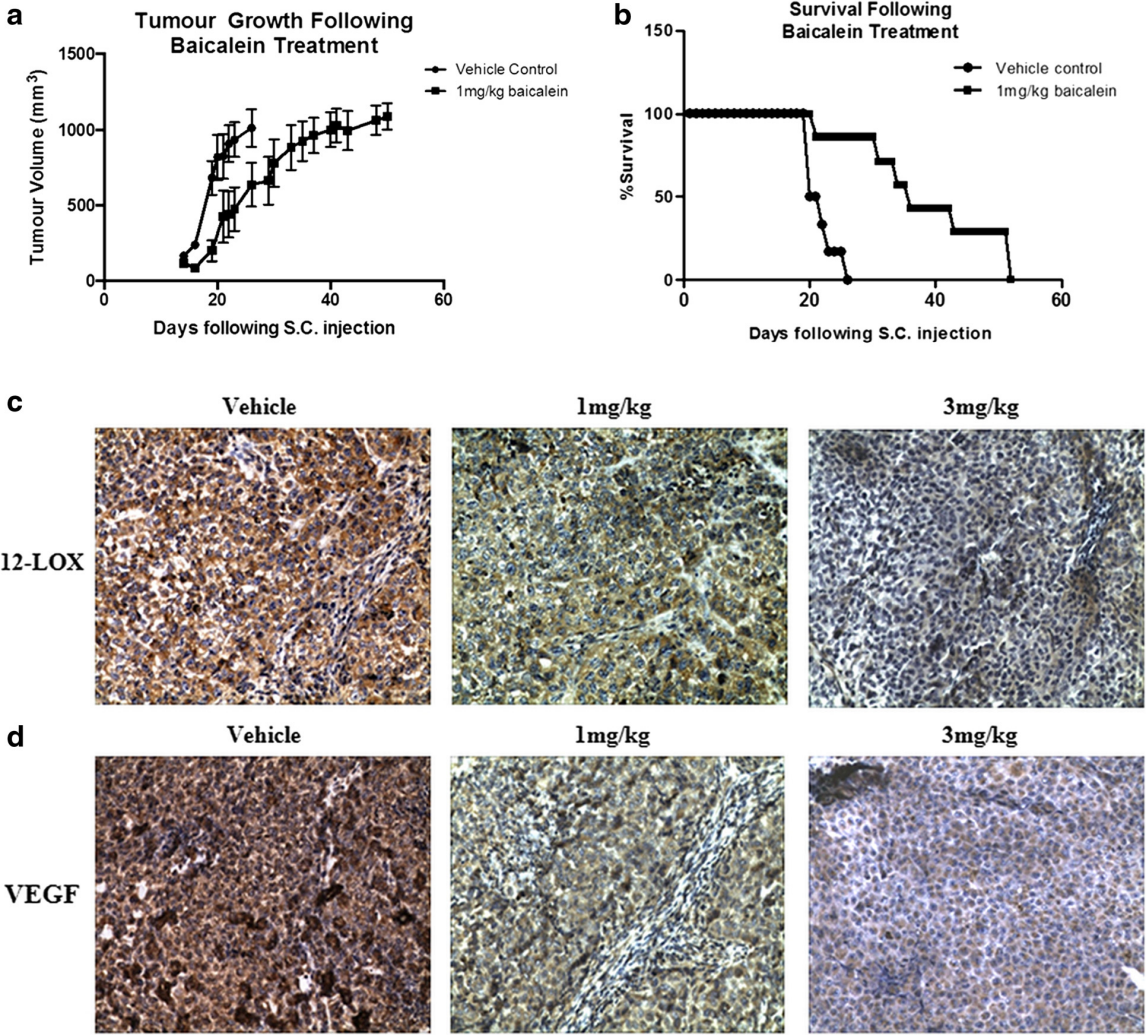
Effect of baicalein treatment on NSCLC tumour growth in-vivo. A xenograft mouse model was generated using H-460 NSCLC cells. When tumour size reached approximately 50 mm^3^, animals were randomised into control and treatment groups (*n* = 7/group). Mice were administered either the flavanoid, baicalein (dissolved in 50 μl DMSO/PBS), or an equal volume of a vehicle control (20 % DMSO in PBS), by intra-tumoural injection (twice weekly). Baicalein treatment significantly reduced tumour growth, relative to vehicle-treated controls (**a**; *n* = 7/group, *p < 0.05). Treatment also prolonged survival of these xenograft mice (**b**). Immunohistochemical staining of the xenograft tumour tissue revealed reduced 12-LOX expression following baicalein treatment (**c**), while VEGF expression was also negatively affected (**d**)

The sub-cutaneous (s.c.) xenograft mouse model of tumour growth was used to examine a potential role for baicalein in the treatment of NSCLC in-vivo. All (21/21) experimental animals were used in the subsequent analysis. Monitoring of tumour growth for approximately 4 weeks post-injection revealed a significant (*p* < 0.05) reduction in growth (as determined by measurement of tumour volume, described above) in baicalein-treated H-460 tumours, relative to PBS + DMSO treated controls (*n* = 7/group; Fig. 4a). This was paralleled by a considerable reduction in animal survival (animals were sacrificed once the tumours reached a size of 1500 mm in any direction;*n* = 7/group; Fig. 4b). Median survival (following first baicalein treatment) was 13 days for the vehicle control group, relative to a median survival of 26 days for the baicalein-treated group. All mice in the vehicle control group were sacrificed by day 26, while almost 30 % of baicalein-treated mice survived for 52 days (86 % survival on day 26). Baicalein was well tolerated in all mice treated with the drug, with no significant difference in animal weight observed during the course of treatment. Notably, the higher concentration of baicalein 3 mg/Kg did not extend survival further in the sub-cutaneous (s.c.) xenograft mouse model. In fact, while tumour growth was inhibited and survival was significantly extended in these mice (Additional file 2: Figure S2), the higher dose of baicaline was less effective that then 1 mg/kg dose. This is most likely due to baicalein inducing a greater innate immune response following higher rates of apoptosis in the tumours, which could have resulted in more immune infiltrate and larger tumour bulk, resulting in the animals being sacrificed earlier when the tumours reached the 1500 mm^3^ size.

**Fig. 5:**
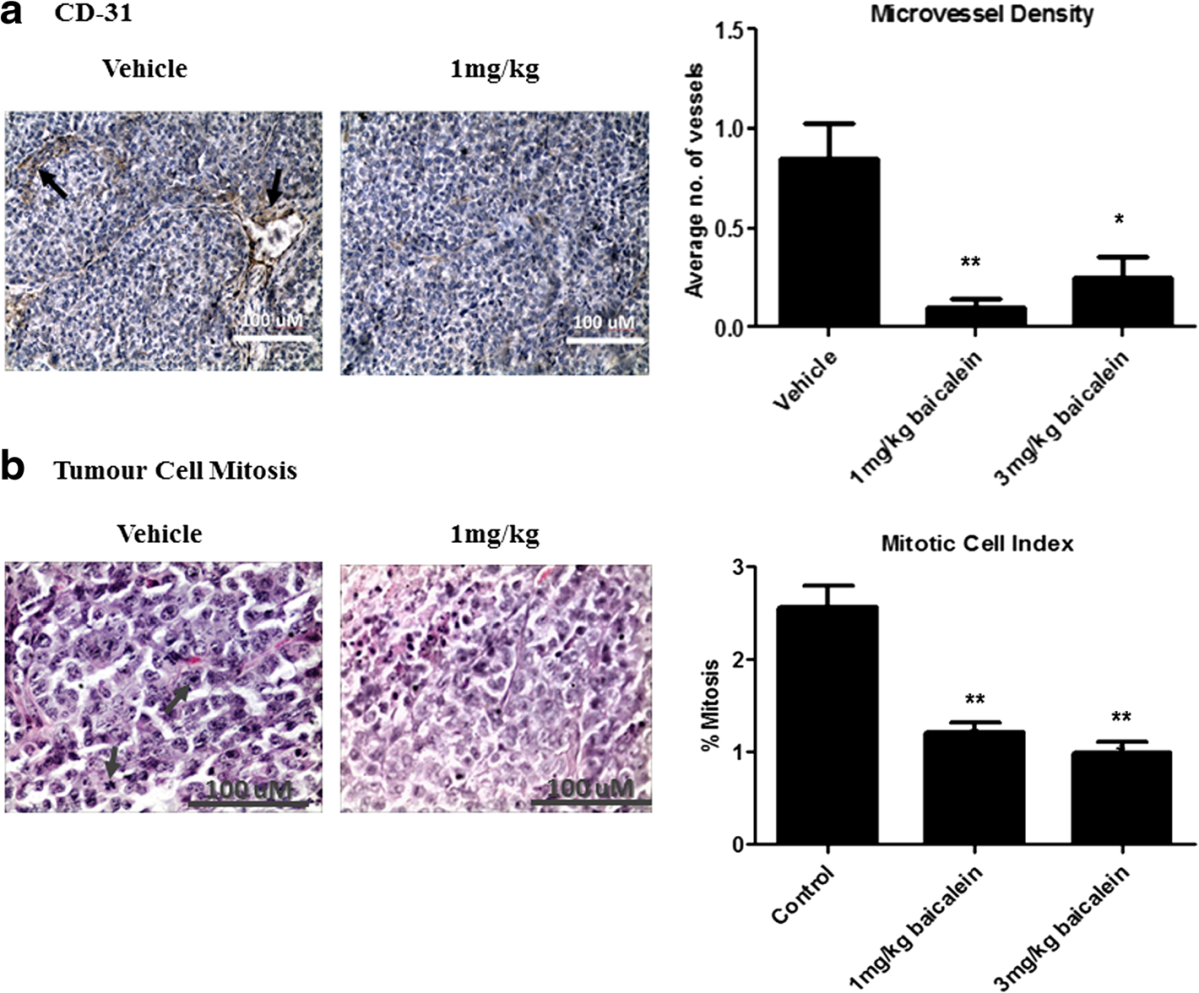
In-vivo tumour cell growth and angiogenesis following baicalein treatment. Tumour tissue from all xenografts was processed for histological analysis. Immunohistochemical staining revealed an increase in mitotic index (**a**), coupled with a reduction in microvessel density (**b**) following treatment. Data is expressed as mean ± SEM (*n* = 7/group; *p < 0.05, ***p < 0.01)

Histologic examination demonstrated reduced 12-LOX (Fig. 4c) and VEGF (Fig. 4d) expression in the baicalein-treated xenograft groups, relative to the saline-treated controls. This was paralleled by a significant reduction in mitotic cell index (1.21 % ± 0.1, 1 mg/kg baicalein *vs*. 2.6 % ± 0.23 control; *p* < 0.001; 0.99 % ± 0.12, 3 mg/kg baicalein *vs*. 2.6 % ± 0.23, control; *p* < 0.0001; *n* = 7/group; Fig. 5a). Microvessel density was also significantly reduced by baicalein treatment (*p* < 0.01, 1 mg/kg baicalein *vs*. control; *p* <0.01, 3 mg/kg baicalein *vs*. control; *n* = 7/group), as indicated by CD-31 staining (Fig. 5b).

### Baicalein-induced changes in gene expression in-vivo

Human Cancer Pathway Finder RT^2^ PCR *Profiler*™ PCR arrays were incorporated to determine the molecular mechanisms underlying the effects of baicalein treatment on tumour growth and survival in-vivo. RNA was isolated from baicalein-treated (1 mg/kg) H-460 xenografts and corresponding vehicle treated controls (*n* = 2/group). First strand cDNA was synthesized from 1 μg of each RNA sample and used for gene-expression analysis. Array data was pooled from 2 mice/group and used to generate a gene-expression profile following treatment.

A number of genes were differentially regulated (greater than 2-fold increase or decrease) in the baicalein-treated group, relative to the vehicle control group (Table 1). A total of eleven genes were significantly altered following baicalein treatment, with gene expression changes across all biological pathways observed. The greatest number of gene-changes were observed in the cell cycle control and DNA damage repair pathway (3 genes), followed by adhesion, angiogenesis, and invasion and metastasis pathways (2 genes altered in each pathway). The most significantly up-regulated genes included *TNFRSF25* (+3.4) and *ITGB3* (+6.96), which have been shown to induce apoptosis, and have been associated with increased survival in cancer. The most significantly down-regulated gene was *FGFR2* (−8.27).

**Table 1:** Effect of baicalein treatment on cancer gene expression in-vivo. Genes shown to be up-regulated or down-regulated in H-460 cells following baicalein treatment, by qPCR. RNA was extracted from xenograft tumour tissue treated with baicalein and corresponding control tissue (*n* = 2/group). cDNA was prepared from this RNA and gene expression profiling carried out using Taqman quantitative PCR arrays (Cancer Profiler Arrays, Superarray). 11 genes were found to be differentially regulated (greater than 2 fold increase/decrease) in the baicalein-treated tumours, relative to vehicle-treated controls

Altered gene	Gene name	Function	Fold change
CDC25A	Cell division cycle 25A	Cell cycle arrest. Allows time for DNA repair.	−2.79
CHEK2	Checkpoint kinase 2	Cell cycle check-point regulator and tumor suppressor	−2.24
E2F1	E2F transcription factor 1	Cell cycle control. Mediator of P53 - dependant apoptosis.	+2.27
TNFRSF25 (DR3)	TNF receptor superfamily; member 25	Increases apoptosis. Anti-metastatic.	+3.4
ERBB2	V-ERB-B2 avian erythroblastic leukemia viral oncogene homolog 2	Oncogene. Mutations associated with lung cancer.	−2.16
ITGA1	Integrin alpha-1	Involved in cell-cell adhesion.	−2.11
ITGB3	Integrin beta-3	Cell adhesion and cell surface mediated signalling. Involvedin platelet aggregation.	+6.96
FGFR-2	Fibroblast Growth factor R2	Angiogenic receptor. Inhibition blocks small cell lung cancer growth in-vitro and in-vivo.	−8.27
IFNB-1	Interferon B1	Anti-tumor effects.	−2.07
MMP-9	Matrix metalloproteinase-9	Invasion of tumour cells through basement membrane. Implicated in lung metastasis of breast tumours.	+2.08
PLAU	Urinary plasminogen activator	Converts plasminogen to plasmin. Stimulates cell migration.	+2.04

**Fig. 6:**
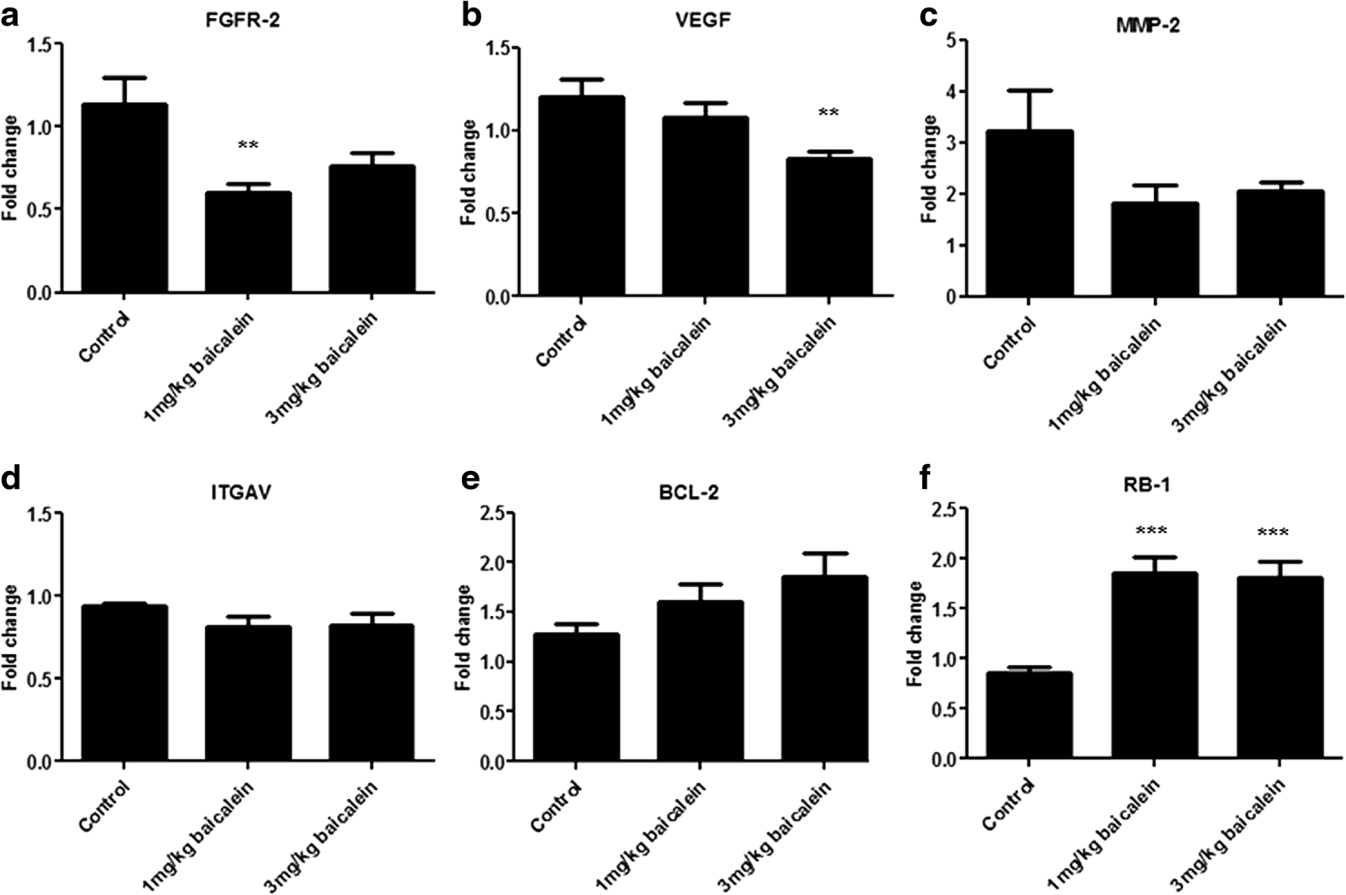
Gene expression profiling following baicalein treatment. A panel of genes were selected for gene expression analysis by quantitative real-time PCR using specific probe/primer sets. *FGFR-2* (**a**) was the most significantly altered gene to come out of the PCR arrays (Table 1). *VEGF* (**b**), *MMP-2* (**c**) and *ITGAV* (**d**) have been implicated in tumour angiogenesis and were also selected based on previous observations in the literature. *Bcl-2* (**e**) has been implicated in the apoptotic response to baicalein, while *RB-1* (**f**) is a known lung cancer gene. Data is expressed as mean ± SEM (*n* = 7/group; **p < 0.01, ***p < 0.0001)

The most significantly altered gene on this array (*FGFR2*) was selected for further validation studies. A further panel of genes was also selected based on previous observations in the literature. This panel was mainly comprised of genes implicated in angiogenesis and apoptosis pathways and included *VEGFA*, *FGFR2*, *Bcl-2*, *ITGAV*, *RB-1, MMP-2*, *MMP-9*, *IGF-1* and *Ang-2*. No amplification of *IGF-1* or *Ang-2* was observed (data not shown) suggesting that these genes are expressed at a very low level in H-460 xenografts. Expression of both *FGFR-2* and *VEGF* was significantly (*p* < 0.01) reduced by baicalein treatment, relative to the control group (Fig. 6a and b), validating previous observations in-vivo and in-vitro. MMP-2 and MMP-9 have previously been shown to be negatively affected by baicalein treatment [10, 16]. A reduction in *MMP-9* expression following baicalein treatment was not observed in this study, although a trend towards reduced *MMP-2* expression was observed following treatment (*p* = 0.14; 1.8 ± 0.3 baicalein treated *vs.* 3.2 ± 0.8 vehicle control; Fig. 6c). There was no significant difference in *ITGAV* levels between control and treatment groups (Fig. 6d). *Bcl-2* levels appeared to increase, although this failed to reach statistical significance (Fig. 6e). *RB-1* (a tumour suppressor gene, which regulates cell survival and cell death) was significantly (*p* < 0.001) increased by baicalein treatment at both concentrations (Fig. 6f).

To determine if these gene alterations are a more generalised effect of baicalein in NSCLC, two other NSCLC cell lines, A549 and SKMES1, were treated with baicalein and gene changes were determined using the same arrays. Notably in both A549 and SKMES1 cells, a number of similar genes were altered (Additional file 3: Table S1 and Additional file 4: Table S2). In the SKMES1 cell line the validated gene FGFR-2 was downregulated 3.29 fold following treatment with baicalein. Additionally gene levels of VEGF were decreased in both the A549 (2.55 fold) and the SKMES1 (3.39 fold), indicating a generalised effect of baicalein on angiogenic gene expression profiles across at least three different NSCLC cell lines. Angiogenic regulators formed the highest group of altered genes when grouped according to cancer hallmark, with 28 % of altered genes in A549 and 33 % of altered genes in the SKMES1 cell line. A decrease in integrin expression was also seen in SKMES1 cells, with ITGA2 and ITGA4 being decreased by 2.09 and 28–fold respectively. While ITGA1 was decreased (2.11 fold) by baicalein in the H460 tumours, this indicates a common effect of baicalein on integrin alpha expression across a panel of NSCLC cells.

## Discussion

Baicalein is a bioactive flavonoid originally isolated from the roots of *Scutellaria baicalensis*. The flavonoid has been shown to inhibit certain types of lipoxygenases [17] and also acts as an anti-inflammatory agent [18]. It has demonstrated considerable promise as an anti-cancer agent both in-vitro [5, 9, 14, 19] and in-vivo [12, 13, 20, 21]. While some investigators have used this agent as a target of the LOX pathway in cancer [14, 22], more recent studies have focused on the anti-cancer effects of this compound and elucidating the mechanisms underlying these effects. While numerous in-vitro studies have been carried out with baicalein in a range of cancer types, the relative number of in-vivo studies with this agent is small, with its in-vivo efficacy in NSCLC not reported. In light of promising in-vitro data in NSCLC, the aim of this study was to investigate the role of baicalein as an anti-cancer agent in-vivo in NSCLC and to uncover potential mechanisms underlying these effects. Our study demonstrates that baicalein reduces growth and improves survival in-vivo, an effect that is at least partly mediated through effects on cell cycle and tumour angiogenesis.

Using a number of in-vitro assays, we first demonstrated the anti-proliferative and pro-apoptotic effects of baicalein in the H-460 cell line. A dose-dependent reduction in cell proliferation was observed following baicalein treatment and this was validated by High Content Screening. This decrease in cell numbers was associated with an increase in apoptosis, confirming initial observations by Leung et al. [14]. Using a fluorochrome based multi-parameter apoptosis assay we observed a significant increase in nuclear condensation and mitochondrial activity, in conjunction with a significant loss of cytoskeletal integrity and the formation of apoptotic bodies. Qualitative observations were validated by quantification using the In Cell Analyser. While these observations are merely a snap-shot of cellular structure at a selected time-point, they indicate significant changes in many characteristics of apoptosis following baicalein treatment. Similar anti-proliferative and pro-apoptotic effects have been observed in pancreatic and prostate cancer cells following baicalein treatment [6, 22]. Zhang et al., demonstrated the growth inhibitory and pro-apoptotic effects of baicalein treatment in oesophageal squamous cell carcinoma cells. They demonstrated increased expression of pro-apoptotic mediators’ caspase-9 and −3 as well as PARP following treatment. They also found components of the PI3K/Akt pathway to be upregulated by baicalein [13]. Baicalein treatment of colon cancer cells inhibited cell growth and induced apoptotic cell death [8]. The authors demonstrated that apoptosis induction was associated with cleavage of poly(ADP-ribose) polymerase, while NF-kB was suppressed through PPARγ activation. Our study did not assess the molecular mechanisms underlying baicalein-mediated effects in-vitro, but instead used a xenograft mouse model to examine the anti-tumour effects and mechanisms of this agent in-vivo.

Treatment of H-460 xenografts with baicalein (intra-tumoural injection) resulted in a significant decline in tumour growth and increased survival in-vivo. Subsequent histological analysis of xenograft tumours revealed a significant loss in mitosis (mitotic index) and a corresponding reduction in angiogenesis (microvessel density). While there are some limitations associated with this experimental approach (using a homogeneous tumour cell population derived from humans to inject into mice), a similar approach has been used to test the in-vivo efficacy of baicalein in other cancer types. While ours is the first study to demonstrate a growth-inhibitory effect of baicalein in lung tumours, a similar effect was previously observed following oral baicalein administration in prostate tumours, confirming our observations [12]. Anti-proliferative and anti-angiogenic (sprout assay) effects were also demonstrated in prostate cancer cell lines, which is in further agreement with our study. The incidence of colitis-associated colon tumour formation (induced by azoxymethane and dextran sulphate sodium) was also significantly reduced by baicalein treatment, supporting our own observations [13]. Several reports have demonstrated that the anti-proliferative effects of baicalein are mediated *via* its inhibitory action on 12-LOX [23, 24]. It was originally demonstrated to be a selective inhibitor of 12-lipoxygenase (12-LOX), although it has more recently also been shown to inhibit the activity of reticulocyte human 15-LOX-1, which is highly expressed in malignant cancer cells [17]. LOXs have been shown to regulate cell survival and apoptosis in a number of cell types [25]. We observed reduced 12-LOX protein expression in baicalein-treated xenograft tissue (relative to vehicle-control tissue) following histological analysis, providing evidence for the in-vivo activity of this agent. We also observed reduced VEGF expression in the treated tissue providing support for an anti-angiogenic mechanism of action of baicalein.

The molecular mechanisms underlying the effects of baicalein in-vivo in NSCLC have not yet been elucidated. In light of this, we used low-density gene-expression arrays (Cancer Pathway Profiler Arrays) to quantitatively assess the effect of baicalein on a panel of 84 genes associated with the hallmarks of cancer. Notable changes in genes involved in the pathways of cell cycle control, apoptosis, adhesion, angiogenesis, and invasion/metastasis were observed following baicalein treatment. While baicalein significantly reduced tumor growth and survival in-vivo, its effect on gene-expression patterns were modest. Only 11 genes were altered by greater than 2-fold following treatment and just 3 genes were altered by greater than 3-fold. This is likely due to the relatively low concentration of baicalein used in our study, although the significant effect on tumour growth and subsequent histological features demonstrate its benefit. With the exception of Kim et al., previous xenograft studies with baicalein have used higher concentrations, although none of these have carried out gene or protein expression analysis on the resulting tumour tissue [12, 13, 20, 21]. Additionally, previous studies have been cell-line based and therefore failed to assess the contribution of the tumour microenvironment.

As baicalein reduced angiogenesis and VEGF protein expression in our xenograft tissue, we initially focused on angiogenic genes and validated genes changes using qPCR probe assays (using all samples). *FGFR-2* was the most significantly down-regulated gene. FGFR-2 protein has been reported to be over-expressed in NSCLC [26], while FGFR inhibition has recently been shown to block lung cancer growth both in-vitro and in-vivo [27, 28]. While VEGF gene-expression was not significantly altered following 1 mg/kg baicalein, a significant reduction in expression was observed following 3 mg/kg treatment. VEGF is a known potent angiogenic factor, which has previously been shown to be negatively affected by baicalein treatment [29]. The anti-angiogenic effect of baicalein has previously been reported in HUVECs, where it significantly reduced the angiogenic response induced by VEGF in a CAM assay. Tubule formation was also reduced following baicalein treatment and MMP-2 activity reduced [10]. Expression of both *MMP-2* and *MMP-9* were assessed in our study following baicalein treatment. Both have important roles in degradation of the basement membrane and are also involved in tumour cell invasion and metastasis [27]. A decrease in *MMP-2* was observed in our study although this failed to reach statistical significance. The effect of baicalein on angiogenic gene expression in lung cancer is further supported by data from two other NSCLC subtypes, both of which displayed a great number of gene alterations focused on angiogenesis and the VEGF signalling pathway following treatment with baicalein. Notably *FGFR2*, *VEGF*, *MMP1*, *TEK* and *ANGPT2* were all downregulated in the squamous NSCLC line SKMES1. Similarly, *VEGF*, *PDGFB*, *TGFBR*, *TEK* and *ANGPT2* were all downregulated in A549 cells indicating many overlapping angiogenic targets effected by baicalein.

A second subset of genes regulating cell cycle was also altered following treatment with baicalein. As mitotic index was reduced in our xenograft tissues, we validated a number of these genes by qPCR. Baicalein was previously reported to effect cell survival in prostate cancer by arresting the cell cycle during the G0/G1 phase [6], whereas in the lung cancer line H-460, the arrest was found to be at the S-phase [14]. We have previously demonstrated that baicalein induced cell cycle arrest in prostate cancer cell lines [6]. In this study we observed altered expressionof a number of genes implicated in cell cycle (*CDC25A*, *CHEK-2*, *E2F-1*) and apoptosis (*TNFRSF25*), following treatment in-vivo.

An up-regulation of the transcription factor *E2F1* has been observed in tumor explants following baicalein treatment. This increased expression is likely due to a significant upregulation of the retinoblastoma-1 (*RB-1*) tumour suppressor gene that we observed following baicalin treatment (validation cohort). *RB-1* is commonly mutated in NSCLC [15] and its main function is in the control of cell growth, through binding to and sequestering the transcriptional activity of the *E2F1* transcription factor. We have previously shown that baicalein inhibits the phosphorylation of RB protein in prostate cancer cell lines, which is associated with the release of E2F [6].

## Conclusions

This is the first study to demonstrate a growth inhibitory and pro-survival effect for baicalein in-vivo in NSCLC. This study also uncovers histological mechanisms associated with baicalein treatment in-vivo, including inhibitory effects on cell proliferation and tumour angiogenesis. At the protein level, both 12-LOX and VEGF expression were reduced by baicalein treatment, while at the gene level significant alterations in expression were observed for *VEGF*, *FGFR-2* and *RB-1*. It is likely that the growth inhibitory effects are mediated in part through RB-1, while the anti-angiogenic effects may be partly mediated *via* VEGF and FGFR-2. Baicalein may therefore have therapeutic efficacy in NSCLC and warrants further investigation.

## Additional files

Additional file 1: Figure S1. Effect of baicalein treatment on lung tumour cell proliferation/survival in A549 and SKMES1 cell lines. Tumour cell proliferation was assessed following 24 h treatment (10 nM, 100 nM, 1 μM and 10 μM baicalein) by BrdU assay. Baicalein treatment resulted in a significant reduction in tumour cell survival in both the A549 (a) and SKMES1 cells (b). Data is expressed as mean ± SEM of three independent experiments, with cell proliferation expressed as a percentage of untreated controls (**p* < 0.05, ***p* < 0.01). (TIFF 49 kb) Additional file 2: Figure S2. Effect of 3 mg/kg baicalein treatment on NSCLC tumour growth in-vivo. A xenograft mouse model was generated using H-460 NSCLC cells. When tumour size reached approximately 50 mm^3^, animals were randomised into control and treatment groups (*n* = 7/group). Mice were administered either the 3 mg/Kg baicalein (dissolved in 50 μl DMSO/PBS), or an equal volume of a vehicle control (20 % DMSO in PBS), by intra-tumoural injection (twice weekly). Baicalein treatment significantly prolonged survival of these xenograft mice relative to vehicle-treated controls (*n* = 7/group, **p* < 0.05). However no additional survival was observed compared to the lower 1 mg/Kg dose. (TIFF 70 kb) Additional file 3: Table S1. Effect of baicalein treatment on cancer gene expression in the A549 cell line in-vitro. RNA was extracted from A549 cells following 1 μM baicalein treatment and corresponding control A549 cells (*n* = 2). cDNA was prepared from this RNA and gene expression profiling carried out using Taqman quantitative PCR arrays (Cancer Profiler Arrays, Superarray). Genes listed were found to be differentially regulated (greater than 2 fold increase/decrease) in the baicalein-treated tumour cells, relative to vehicle-treated controls. (TIFF 72 kb) Additional file 4: Table S2. Effect of baicalein treatment on cancer gene expression in the SKMES1 cell line in-vitro. RNA was extracted from SKMES1 cells following 1 μM baicalein treatment and corresponding control SKMES1 cells (*n* = 2). cDNA was prepared from this RNA and gene expression profiling carried out using Taqman quantitative PCR arrays (Cancer Profiler Arrays, Superarray). Genes listed were found to be differentially regulated (greater than 2 fold increase/decrease) in the baicalein-treated tumour cells, relative to vehicle-treated controls. (TIFF 73 kb)

## Abbreviations

H and E: Haemytoxylin and Eosin
IHC: Immunohistochemistry
NSCLC: Non-small cell lung cancer
SC: Subcutaneous.
