# Supplementary figures and images for: Anti-cancer effects of baicalein in non-small cell lung cancer in-vitro and in-vivo
Source: BMC Cancer. 2016 Sep 1;16(1):707. doi: 10.1186/s12885-016-2740-0 (PMC5009689; doi:10.1186/s12885-016-2740-0)

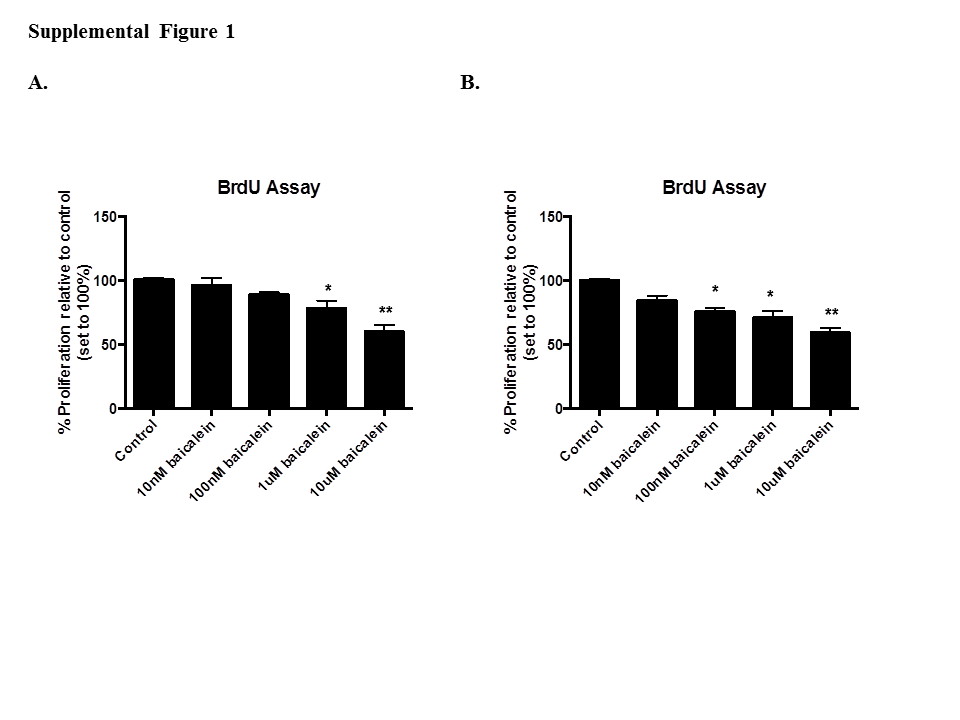

Supplement: Additional file 1: Figure S1. — Effect of baicalein treatment on lung tumour cell proliferation/survival in A549 and SKMES1 cell lines. Tumour cell proliferation was assessed following 24 h treatment (10 nM, 100 nM, 1 μM and 10 μM baicalein) by BrdU assay. Baicalein treatment resulted in a significant reduction in tumour cell survival in both the A549 (a) and SKMES1 cells (b). Data is expressed as mean ± SEM of three independent experiments, with cell proliferation expressed as a percentage of untreated controls (*p < 0.05, **p < 0.01). (TIFF 49 kb) [file 12885_2016_2740_MOESM1_ESM.tiff]

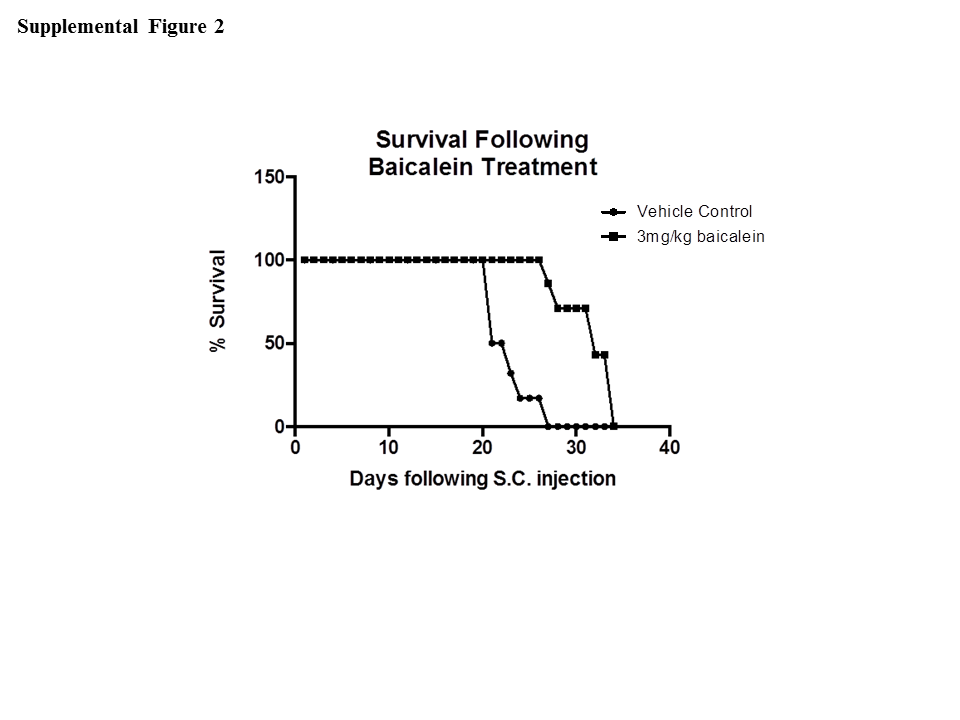

Supplement: Additional file 2: Figure S2. — Effect of 3 mg/kg baicalein treatment on NSCLC tumour growth in-vivo. A xenograft mouse model was generated using H-460 NSCLC cells. When tumour size reached approximately 50 mm3, animals were randomised into control and treatment groups (n = 7/group). Mice were administered either the 3 mg/Kg baicalein (dissolved in 50 μl DMSO/PBS), or an equal volume of a vehicle control (20 % DMSO in PBS), by intra-tumoural injection (twice weekly). Baicalein treatment significantly prolonged survival of these xenograft mice relative to vehicle-treated controls (n = 7/group, *p < 0.05). However no additional survival was observed compared to the lower 1 mg/Kg dose. (TIFF 70 kb) [file 12885_2016_2740_MOESM2_ESM.tiff]

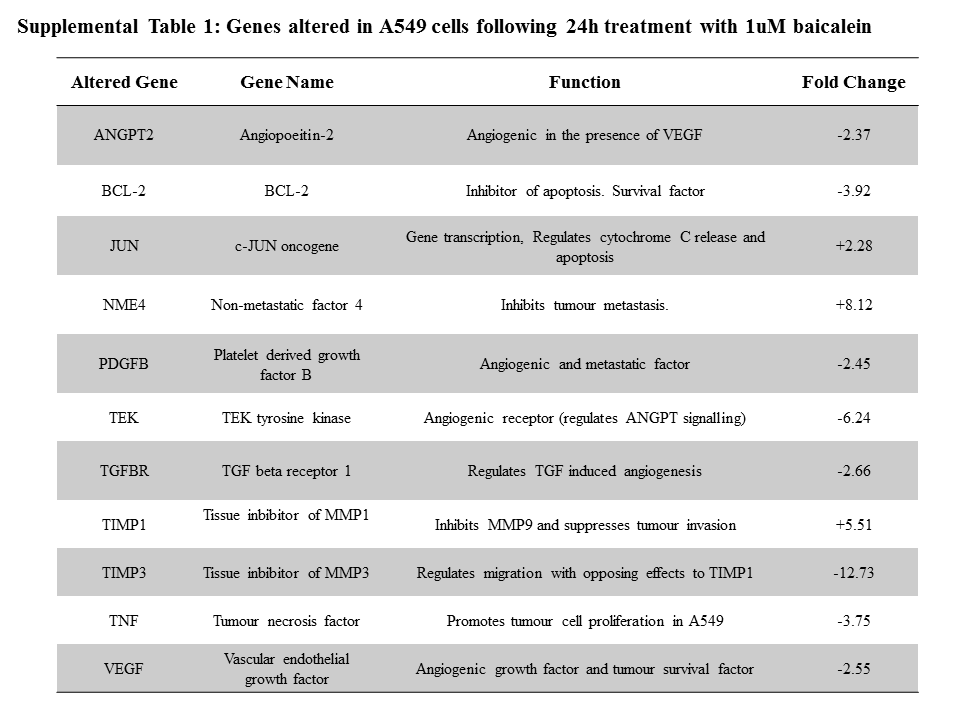

Supplement: Additional file 3: Table S1. — Effect of baicalein treatment on cancer gene expression in the A549 cell line in-vitro. RNA was extracted from A549 cells following 1 μM baicalein treatment and corresponding control A549 cells (n = 2). cDNA was prepared from this RNA and gene expression profiling carried out using Taqman quantitative PCR arrays (Cancer Profiler Arrays, Superarray). Genes listed were found to be differentially regulated (greater than 2 fold increase/decrease) in the baicalein-treated tumour cells, relative to vehicle-treated controls. (TIFF 72 kb) [file 12885_2016_2740_MOESM3_ESM.tiff]

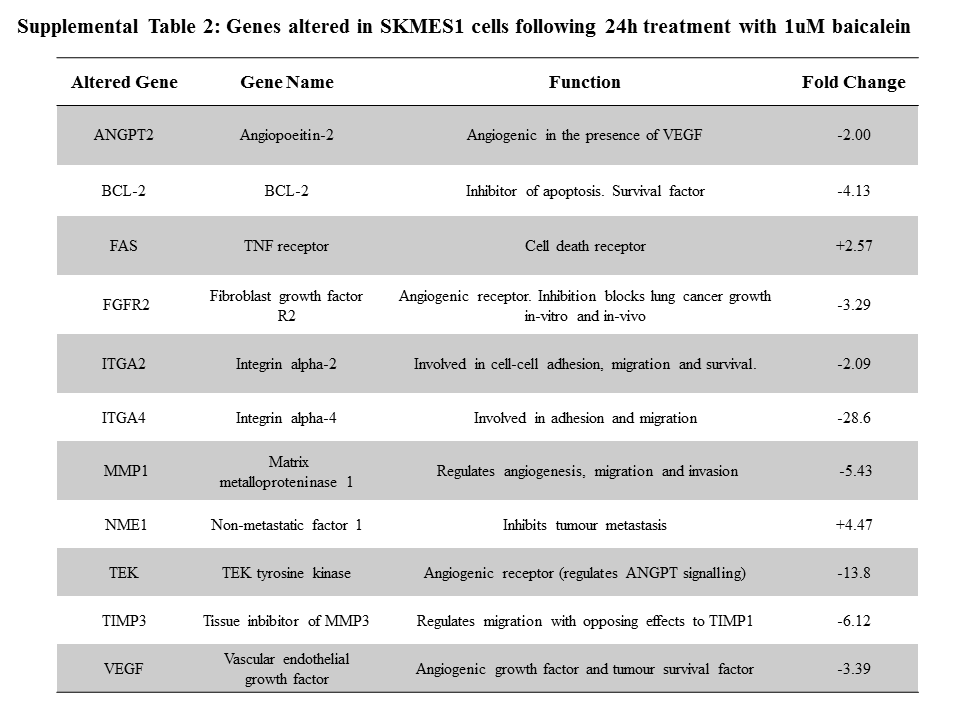

Supplement: Additional file 4: Table S2. — Effect of baicalein treatment on cancer gene expression in the SKMES1 cell line in-vitro. RNA was extracted from SKMES1 cells following 1 μM baicalein treatment and corresponding control SKMES1 cells (n = 2). cDNA was prepared from this RNA and gene expression profiling carried out using Taqman quantitative PCR arrays (Cancer Profiler Arrays, Superarray). Genes listed were found to be differentially regulated (greater than 2 fold increase/decrease) in the baicalein-treated tumour cells, relative to vehicle-treated controls. (TIFF 73 kb) [file 12885_2016_2740_MOESM4_ESM.tiff]
